# High nutritional risk is associated with unfavorable outcomes in patients admitted to an intensive care unit

**DOI:** 10.5935/0103-507X.20190041

**Published:** 2019

**Authors:** Julia Marchetti, Audrey Machado dos Reis, Amanda Forte dos Santos, Oellen Stuani Franzosi, Vivian Cristine Luft, Thais Steemburgo

**Affiliations:** 1 Departamento de Nutrição, Faculdade de Medicina, Universidade Federal do Rio Grande do Sul - Porto Alegre (RS), Brasil.; 2 Programa de Pós-Graduação em Alimentos, Nutrição e Saúde, Universidade Federal do Rio Grande do Sul - Porto Alegre (RS), Brasil.; 3 Residência Multidisciplinar Integrada em Saúde, com Ênfase em Terapia Intensiva de Adultos, Hospital de Clínicas de Porto Alegre, Universidade Federal do Rio Grande do Sul - Porto Alegre (RS), Brasil.; 4 Centro de Estudos em Alimentos e Nutrição, Hospital de Clínicas de Porto Alegre, Universidade Federal do Rio Grande do Sul - Porto Alegre (RS), Brasil.

**Keywords:** Nutrition assessment, Nutritional status, Risk factors, Critical care, Intensive care units

## Abstract

**Objective:**

To evaluate possible associations between nutritional risk and the clinical outcomes of critical patients admitted to an intensive care unit.

**Methods:**

A prospective study was carried out with a cohort comprising 200 patients admitted to a university hospital intensive care unit. Nutritional risk was assessed with the NRS-2002 and NUTRIC scores. Patients with scores ≥ 5 were considered at high nutritional risk. Clinical data and outcome measures were obtained from patients' medical records. Multiple logistic regression analysis was used to calculate odds ratios and their respective 95% confidence intervals (for clinical outcomes).

**Results:**

This sample of critical patients had a mean age of 59.4 ± 16.5 years and 53.5% were female. The proportions at high nutritional risk according to NRS-2002 and NUTRIC were 55% and 36.5%, respectively. Multiple logistic regression models adjusted for gender and type of admission indicated that high nutritional risk assessed by the NRS-2002 was positively associated with use of mechanical ventilation (OR = 2.34; 95%CI 1.31 - 4.19; p = 0.004); presence of infection (OR = 2.21; 95%CI 1.24 - 3.94; p = 0.007), and death (OR = 1.86; 95%CI 1.01 - 3.41; p = 0.045). When evaluated by NUTRIC, nutritional risk was associated with renal replacement therapy (OR = 2.10; 95%CI 1.02 - 4.15; p = 0.040) and death (OR = 3.48; 95%CI 1.88 - 6.44; p < 0.001).

**Conclusion:**

In critically ill patients, high nutritional risk was positively associated with an increased risk of clinical outcomes including hospital death.

## INTRODUCTION

Malnutrition is a frequent condition among hospitalized individuals.^(1,2)^ It is even more prevalent among critically ill patients admitted to intensive care units (ICUs), considering that they are often in a hypermetabolic state caused by trauma or stress from the acute disease.^(3,4)^ It is known that malnutrition is associated with poor clinical outcomes, such as increased morbidity and mortality, longer hospital stays, and clinical complications, which result in higher costs for health systems.^(2,5)^

One of the most effective strategies for management of high nutritional risk patients is to implement specialized nutritional interventions.^(6-10)^ In these patients, identifying nutritional risk as quickly as possible may help to ensure nutritional therapy is initiated earlier. Such measures are essential to reduce adverse events and improve these patients' quality of life during hospitalization and recovery.^(11)^

Identification of nutritional risk in critically ill patients is a challenge for healthcare professionals because each nutritional screening tool has its limitations and specific characteristics. As a result, there is no international consensus that establishes which is the best tool for assessing nutritional risk in this population. The Nutritional Risk Screening 2002 (NRS-2002)^(12)^ and the Nutrition Risk in the Critically Ill (NUTRIC)^(6)^ scores appear to be the most adequate tools for screening such patients because they consider their nutritional condition and the impact of the disease or trauma on nutritional status.^(13)^

The NRS-2002 is recommended by the European Society for Clinical Nutrition and Metabolism (ESPEN), was the first screening tool developed using evidence-based medicine, and can be administered to all hospitalized patients.^(12)^ This tool identified high nutritional risk in 40% of a sample of patients admitted to an ICU.^(14)^ Nutritional risk as assessed by NRS-2002 was also associated with mortality and longer hospital stays in ICU patients.^(15,16)^ A recent evaluation of NRS-2002 cut-offs for ICU patients recommended in American Society for Parenteral and Enteral Nutrition (ASPEN) guidelines^(17)^ showed that they were capable of distinguishing between critically ill patients in terms of clinical characteristics and outcomes.^(18)^

On the other hand, the NUTRIC screening tool, validated by Heyland^(19)^ and recommended by ASPEN,^(17)^ was specifically developed to identify nutritional risk in critically ill patients who may benefit from aggressive nutritional therapy. This tool demonstrated that approximately 50% of patients admitted to the ICU are at high nutritional risk.^(20,21)^ Furthermore, in different populations observational studies in critically ill patients in have demonstrated that high nutritional risk identified by NUTRIC is associated with unfavorable clinical outcomes and death.^(15-18,20)^

To date, few studies in Brazil have analyzed associations between high nutritional risk assessed using these tools and clinical outcomes in critical patients. Therefore, the aim of this study was to identify possible associations between high nutritional risk, assessed using these tools, and the clinical outcomes of critically ill patients admitted to an ICU.

## METHODS

A prospective cohort study was performed with a sample of critically ill patients admitted to the ICU at the Hospital de Clínicas de Porto Alegre (HCPA), RS, Brazil. The cohort comprised adult patients (age ≥ 18 years) of both genders, admitted from October 2017 to January 2018. Patients with advanced terminal illness, neurodegenerative diseases, therapeutic limitations, readmitted to the ICU, and pregnant women were excluded from the study.

Patients were selected by daily screening, within a maximum of 72 hours after admission to the ICU. They were followed until hospital discharge or death. All data used in this study were collected from physical and electronic records, from patients, care team, family and/or companions. No modifications were made to patients' treatment while in hospital. The study was conducted according to the Declaration of Helsinki guidelines and all procedures involving patients were approved by the Hospital Ethics Committee (protocol #170524). All patients or their legal guardians signed informed consent forms.

Clinical and demographic characteristics such as age, gender, ethnicity, and type of admission (clinical, surgical, or trauma) were collected from electronic records. Clinical patients were defined as those who had clinical diagnoses with no surgical management; surgical patients were those who had acute abdomen and/or were in perioperative care; and trauma patients were those who had multiple traumatic injuries. Other clinical outcome measures included length of hospital stay (days), length of ICU stay (days), readmission to the ICU, infection during hospitalization, use of mechanical ventilation, mechanical ventilation period (days), use of renal replacement therapy, duration of renal replacement therapy (days), and hospital death. The following infectious complications were considered: urinary, respiratory, and gastrointestinal tract, surgical wounds, central nervous system, and cutaneous infections. All outcomes were obtained from each participant's medical records.

Nutritional screening was conducted by a trained nutritionist using two tools: NRS-2002) (Table S1 - Supplementary material)^(12)^ and NUTRIC (Table S2 - Supplementary material)^(6)^ within 72 hours of admission to the ICU.

The NRS-2002 rates patients' nutritional risk according to five variables: (I) unexplained weight loss in the last three months, (II) appetite, (III) body mass index (BMI), and (IV) disease stress factor. Age (V) over 70 years is considered an additional risk factor.^(12)^

The NUTRIC scale classifies patients according to the following criteria: age, Acute Physiology and Chronic Health Evaluation II (APACHE II) score, Sequential Organ Failure Assessment (SOFA) score, comorbidities, days of hospitalization before admission to the ICU, and interleukin-6 (IL-6). Additionally, in 2015 a study conducted by Rahman et al. revalidated the tool excluding IL-6, since it is not commonly used in clinical practice.^(6)^

### Statistical analysis

Data are presented as mean and standard deviation, median (25th - 75th), or absolute values (%), and compared using Student's *t*, Mann-Whitney U, or χ^2^ tests, respectively. Nutritional risk was evaluated by NRS-2002 and NUTRIC and then classified as tool scores < 5 or ≥ 5 points. Thus, patients with a ≥ 5 score were considered at high nutritional risk. Multiple logistic regression analysis was used to calculate odds ratios (OR) and their respective 95% confidence intervals (95%CIs) for clinical outcomes. All models were adjusted for gender and type of admission. Calculations were performed with the Statistical Package for The Social Sciences (SPSS) 23.0 (Chicago, IL) and p values < 0.05 were considered statistically significant.

## RESULTS

**Figure 1:**
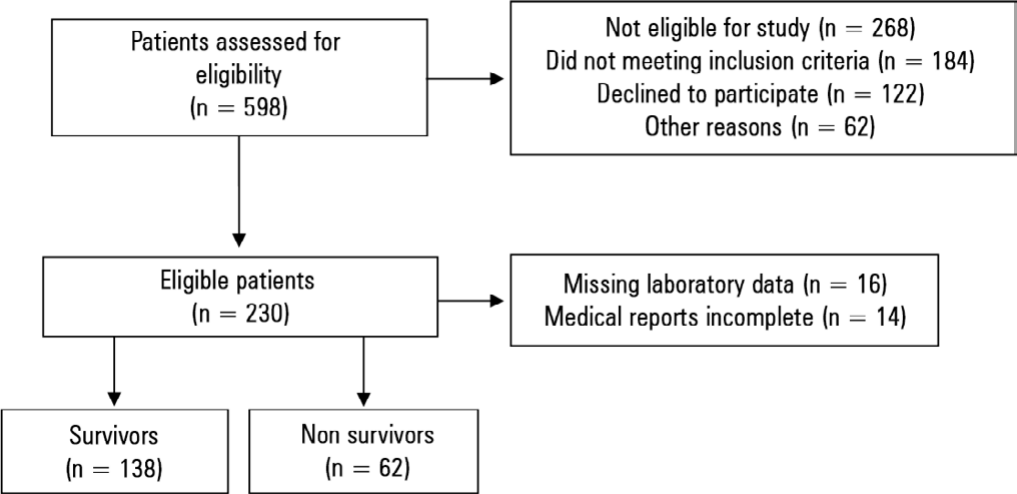
Flowchart of patient selection.

A total of 200 patients were included (59.4 ± 16.5 years old, 53.5% female). The selection process is illustrated in figure 1. The rates of high nutritional risk were 55% (n =110) and 36.5% (n = 73), according to the NRS-2002 and NUTRIC respectively. Furthermore, 25% of the patients (n = 50) were assessed as high nutritional risk by both NRS-2002 and NUTRIC.

The patients' general characteristics are listed in table 1. White ethnicity was reported for 87.5% of the patients and mean BMI was 27.1 ± 8.3Kg/m². With regard to disease severity, mean APACHE II score was 14.7 ± 4.1 and median SOFA score was 5.0 (2.2 - 8.0). More patients were admitted to the ICU with clinical diagnoses (72.5%). Median length of stay (LOS) in hospital was 15.0 days. Median ICU LOS was 4.0 days, and 9.5% of the patients were readmitted to the ICU. With regard to clinical outcomes, 56% of the patients required mechanical ventilation and 20.5% needed renal replacement therapy. Around 50% of the patients had infections while in hospital. The following infections were considered: respiratory tract (28%), urinary tract (12.5%), blood (14%), cutaneous (5%), surgical wound (9%), gastrointestinal (3%), and central nervous system (1%). Overall, 36% of patients admitted to the ICU died.

**Table 1:** General characteristics of critically ill patients admitted to the intensive care unit (n = 200)

	Descriptive statistics
Clinical and demographic data	
Age (years)	59.4 ± 16.5
Sex (female)	93 (53.5)
Ethnicity (white)	175 (87.5)
BMI (kg/m^2^)	27.1 ± 8.3
APACHE II (score)	14.7 ± 4.1
SOFA (score)	5.0 (2.2 - 8.0)
Type of admission	
Clinical	145 (72.5)
Surgical	52 (26)
Trauma	3 (1.5)
Hospitalization and clinical outcomes	
Hospital LOS (days)	15.0 (8.0 - 24.5)
ICU LOS (days)	4.0 (2.0 - 8.0)
Readmission to ICU (yes)	19 (9.5)
Use of mechanical ventilation (yes)	112 (56)
Mechanical ventilation period (days)	3.0 (1.0 - 7.0)
RRT (yes)	41 (20.5)
Period on RRT (days)	8.5 (3.0 - 15.7)
Infection (yes)	100 (50)
Hospital death (%)	62 (36)

BMI - body mass index; APACHE II - Acute Physiology and Chronic Health Evaluation II; SOFA - Sequential Organ Failure Assessment; LOS - length of hospital stay; ICU - intensive care unit; RRT- renal replacement therapy. Results presented as absolute value (%), mean ± standard deviation or median (25^th^ - 75^th^).

Table 2 lists associations between high nutritional risk, evaluated by nutritional screening tools, and clinical outcomes of critically ill patients. Patients with high nutritional risk according to NRS-2002 had associations with prolonged ICU stay, mechanical ventilation use, infection, and death, when compared to patients who had < 5 nutritional risk points. Neither length of hospital stay nor renal replacement therapy had significant associations with high nutritional risk according to NRS-2002. Patients at high nutritional risk according to NUTRIC had associations with renal replacement therapy and death, when compared with other patients. No associations were observed with length of hospital stay, length of ICU stay, or other clinical complications.

**Table 2:** Clinical outcomes of critically ill patients admitted to the intensive care unit according to high nutritional risk

Clinical outcomes	Nutritional screening tools					
	NRS-2002			NUTRIC		
	Score < 5 points (n = 90)	Score ≥ 5 points* (n = 110)	p value	Score < 5 points (n = 127)	Score ≥ 5 points* (n = 73)	p value
Hospital LOS (days)	14.5 (8.0 - 4.2)	16.0 (8.0 - 25.0)	0.433	15.0 (6.0 - 8.0)	14.0 (8.0 - 23.0)	0.700
ICU LOS (days)	3.0 (0.0 - 8.0)	5.0 (0.0 - 8.0)	0.050†	4.0 (2.0 - 8.0)	5.0 (8.0 - 8.5)	0.180
Use of mechanical ventilation (yes)	40 (44.4)	72 (65.5)	< 0.001†	20 (15.7)	31 (28.8)	0.070
RRT (yes)	18 (20.0)	23 (20.9)	0.900	65 (51.2)	47 (64.4)	0.030†
Infection (yes)	35 (17.5)	65 (32.5)	0.004†	63 (31.5)	37 (18.5)	0.500
Hospital death (yes)	25 (27.8)	47 (42.7)	0.003†	32 (25.2)	40 (54.8)	< 0.001†

NRS-2002 - Nutritional Risk Screening - 2002; NUTRIC - Nutrition Risk in the Critically Ill; LOS - length of stay; ICU - intensive care unit; RRT - renal replacement therapy. Results presented as median (25^th^ - 75^th^) or n (%), and compared using Student’s *t*, Mann-Whitney U, and χ2 tests, respectively.

* NResults considered as high nutritional risk.

† statistically significant p values.

Associations with clinical outcomes were evaluated using logistic regression analysis adjusted for gender and type of admission (Table 3). High nutritional risk (score ≥ 5) according to NRS-2002 assessment was positively associated with mechanical ventilation use, presence of infection, and death. Significant and positive associations with high nutritional risk assessed by NUTRIC were also observed with renal replacement therapy and death.

**Table 3:** Multiple logistic regression analysis *high nutritional risks and their odds ratios for clinical outcomes

Nutrition screening tool	MV OR (95%CI)	p value	RRT OR (95%CI)	p value	Infection OR (95%CI)	p value	Hospital death OR (95%CI)	p value
NRS-2002 ≥ 5 points	2.34 (1.31 - 4.19)	0.004†	1.05 (0.52 - 2.13)	0.891	2.21 (1.24 - 3.94)	0.007†	1.86 (1.01 - 3.41)	0.045†
NUTRIC ≥ 5 points	1.65 (0.91 - 3.00)	0.010†	2.10 (1.02 - 4.15)	0.040†	1.08 (0.60 - 1.94)	0.796	3.48 (1.88 - 6.44)	< 0.001†

* All analyses were adjusted for gender and type of admission.

† statistically significant p values.

MV - mechanic ventilation; OR - odds ratio; 95%CI - 95% confidence interval; RRT - renal replacement therapy; NRS-2002 - Nutritional Risk Screening 2002; NUTRIC - Nutrition Risk in the Critically Ill.

## DISCUSSION

In the present study, the prevalence rates of high nutritional risk among critically ill patients were 55% and 36.5%, according to NRS-2002 and NUTRIC respectively. Associations were also observed between high nutritional risk and unfavorable outcomes. These results corroborate previous observational studies that have used these screening tools to identify nutritional risk in ICU patients.^(15,16,20,21)^

In fact, high nutritional risk in critically ill patients is associated with clinical complications such as increased morbidity and mortality, occurrence of infections, and prolonged hospital stay.^(14-16,20)^ In this study, patients at high nutritional risk (score ≥ 5) had higher numbers of days in the ICU and higher rates of mechanical ventilation, infections, and death, when compared to patients with scores < 5. Similar results have been demonstrated in other studies with ICU patients, where a high nutritional risk assessed by NRS-2002 was positively associated with death.^(14-16)^

The prevalence of death observed in this cohort was 36% of patients. When we evaluated death against nutritional risk according to NRS-2002 and NUTRIC, patients with high nutritional risk (> 5) had a higher prevalence of death compared to patients with scores < 5. Additionally, increased risks of mortality (1.86 times) and use of mechanical ventilation (2.34 times) were observed in patients with high nutritional risk according to NRS-2002. In previous studies, high nutritional risk as assessed by NUTRIC was associated with longer hospitalization and clinical complications, such as use of mechanical ventilation and death.^(15,20,21)^ In the current study, we observed that patients at high nutritional risk when assessed by NUTRIC had a greater chance of presence of infection (21%) and renal replacement therapy (10%).

In clinical practice, screening and assessment tools are used to evaluate nutritional status.^(22)^ However, only the NRS-2002 and NUTRIC tools include severity of trauma and/or disease.^(18)^ Although NUTRIC was created specifically for critically ill patients and is a quick and practical assessment tool when patients are unable to communicate, this score has some limitations that should be considered. NUTRIC does not include traditional nutritional risk markers such as BMI, weight loss, food intake, physical examination, or pre-existing malnutrition. Furthermore, there is a lack of criteria related to the period of exposure to high severity disease or trauma (metabolic stress).^(22)^ It is also possible that this tool could be more complex to use in some ICUs, since some biochemical values, such as the marker interleukin-6 (IL-6), are not always available. However, in 2015, Rahman et al. revalidated this tool excluding IL-6,^(6)^ which makes it a better tool in the absence of this biomarker.

NRS-2002 was the first nutritional risk screening tool to be developed using evidence-based medicine.^(12)^ NRS-2002 is effective for identifying patients at high nutritional risk who may benefit from early and aggressive nutritional support.^(7,23,24)^ Indeed, in our study, NRS-2002 identified a 55% prevalence of high nutritional risk among the critically ill patients assessed. Moreover, we observed that patients at high nutritional risk had a higher number of associations with clinical outcomes. It is possible that the screening factors used by this tool identify nutritional risk more specifically. Some important points to consider about NRS-2002 is that all ICU patients with an APACHE score > 10 are considered at nutritional risk, regardless of their nutritional variables. It has been suggested that the APACHE >10 criterion should be replaced by an expectation of an ICU stay lasting at least 1 week (7 days), combined with a need for mechanical ventilation for the same period.^(22)^

Our study has some limitations. First, data for both scores were collected by just one trained investigator. In a study comparing two diagnostic/prognostic models, blinded comparisons would prevent bias. An analysis with a larger number of patients could provide more robust results. Also, this sample comprised patients with a wide age range (including adults and elderly people) and with different diseases. We believe that assessing the nutritional risk according to the different diseases might be interesting and provide more accurate data for nutritional screening of ICU patients. Our sample only included patients who were admitted to the ICU, and our results cannot be extrapolated to all hospitalized patients. Indeed, according to an American study conducted with hospitalized patients,^(25)^ there is still variability in the use of nutritional screening tools. On the other hand, we emphasize that, to date, there had been no studies demonstrating the performance of NUTRIC in relation to NRS-2002 for screening critically ill patients and demonstrating associations between high nutritional risk and clinical outcomes during the period in the ICU (5 days median). Perhaps some results would be more consistent in patients with longer ICU stay, as previously suggested by Kondrup et al.^(22)^

## CONCLUSION

Critically ill patients with high nutritional risk presented increased risks of clinical outcomes, including death. High nutritional risk according to the NRS-2002 score was associated with increased risk of mechanical ventilation, presence of infection, and death. In turn, high nutritional risk according to the NUTRIC score was associated with higher risk of renal replacement therapy and death.
