# Supplementary material for: High nutritional risk is associated with unfavorable outcomes in patients admitted to an intensive care unit
Source: Rev Bras Ter Intensiva. 2019 Jul-Sep;31(3):326–32. doi: 10.5935/0103-507X.20190041 (PMC7005948; doi:10.5935/0103-507X.20190041)
Supplement: Supplementary file 1 [file rbti-31-03-0326-suppl1.pdf]

High nutritional risk is associated with unfavorable outcomes in patients admitted to an intensive care unit

O elevado risco nutricional está associado a desfechos desfavoráveis em pacientes internados na unidade de terapia intensiva

Julia Marchetti[1], Audrey Machado dos Reis[2], Amanda Forte dos Santos[1], Oellen Stuani Franzosi[3,4], Vivian Cristine Luft[1,2,4], Thais Steemburgo[1,2,4]

Table S1 - Nutritional Risk Screening 2002

Nutritional Risk Screening 2002			
**Initial screening**		**Yes**	**No**
1	Is BMI < 20.5?		
2	Has the patient lost weight within the last 3 months?		
3	Has the patient had a reduced dietary intake in the last week?		
4	Is the patient severely ill? (e.g. in intensive therapy)		
Yes: If the answer is 'Yes' to any question, the final screening in is performed.			
No: If the answer is 'No' to all questions, the patient is re-screened at weekly intervals. If the patient e.g. is scheduled for a major operation, a preventive nutritional care plan is considered to avoid the associated risk status.			
**Final screening**			
Impaired nutritional status		Severity of disease (increase in requirements)	
Absent - **score 0**	Normal nutritional status	Absent - **score 0**	Normal nutritional requirements
Mild - **score 1**	Weight loss > 5% in 3 months or Food intake below 50 - 75% of normal requirement in preceding week.	Mild - **score 1**	Hip fracture, Chronic patients, in particular with acute complications: cirrhosis, COPD. Chronic hemodialysis, diabetes, oncology.
Moderate - **score 2**	Weight loss > 5% in 2 months or BMI 18.5 - 20.5 + impaired general condition or Food intake 25 - 60% of normal requirement in preceding week.	Moderate - **score 2**	Major abdominal surgery, Stroke, severe pneumonia, hematologic malignancy.
Severe - **score 3**	Weight loss > 5% in 1 month (> 15% in 3 months) or BMI < 18.5 + impaired general condition or Food intake 0 - 25% of normal requirement in preceding week.	Severe - **score 3**	Head injury, bone marrow transplantation, intensive care patients (APACHE > 10).
**Score:**	**+**	**Score:**	**= Total score**
**Age:**	if ≥ 70 years: add 1 to total score above		**= age-adjusted total score**
**Score ≥ 3:** the patient is nutritionally at-risk and a nutritional care plan is initiated			
**Score < 3:** weekly rescreening of the patient. If the patient e.g. is scheduled for a major operation, a preventive nutritional care plan is considered to avoid the associated risk status.			
**Prototypes for severity of disease:**			
**Score = 1:** a patient with chronic disease, admitted to hospital due to complications. The patient is weak but out of bed regularly. Protein requirement is increased but can be covered by oral diet or supplements in most cases.			
**Score = 2:** a patient confined to bed due to illness, e.g. following major abdominal surgery. Protein requirement is substantially increased, but can be covered, although artificial feeding is required in many cases.			
**Score = 3:** a patient in intensive care with assisted ventilation etc. Protein requirement is increased and cannot be covered even by artificial feeding. Protein breakdown and nitrogen loss can be significantly attenuated.			

BMI - body mass index; COPD - chronic obstructive pulmonary disease; APACHE - Acute Physiology and Chronic Health Evaluation.
Source: Adpted from Kondrup J, Rasmussen HH, Hamberg O, Stanga Z; Ad Hoc ESPEN Working Group. Nutritional risk screening (NRS 2002): a new method based on an analysis of controlled clinical trials. Clin Nutr. 2003;22(3):321-36.[12]

Table S2 - Nutrition Risk in the Critically Ill (NUTRIC)

Table 1. NUTRIC score variables

Variable	Range	Points
**Age**	< 50	0
	50 - 75	1
	≥ 75	2
**APACHE II**	< 15	0
	15 - < 20	1
	20 - 28	2
	≥ 28	3
**SOFA**	< 6	0
	6 - < 10	1
	≥ 10	2
**Number of co-morbidities**	0 - 1	0
	≥ 2	1
**Days from hospital to ICU admission**	0 - <1	0
	≥ 1	1
**IL-6**	0 - < 400	0
	≥ 400	1

Table 2. NUTRIC score scoring system: if IL-6 available

Sum of points	Category	Explanation
6 - 10	High score	Associated with worse clinical outcomes (mortality, ventilation).<br>These patients are the most likely to benefit from aggressive nutrition therapy.
0 - 5	Low score	These patients have a low malnutrition risk.

Table 3. NUTRIC score scoring system: If no IL-6 available*

Sum of points	Category	Explanation
5 - 9	High score	Associated with worse clinical outcomes (mortality, ventilation).<br>These patients are the most likely to benefit from aggressive nutrition therapy.
0 - 4	Low score	These patients have a low malnutrition risk.

APACHE - Acute Physiology and Chronic Health Evaluation; SOFA - Sequential Organ Failure Assessment; ICU - intensive care unit; IL-6 - interleucina-6. * It is acceptable to not include interleucina-6 data when it is not routinely available; it was shown to contribute very little to the overall prediction of the NUTRIC score.

Source: Adpted from Rahman A, Hasan RM, Agarwala R, Martin C, Day AG, Heyland DK. Identifying critically ill patients who will benefit most from nutritional therapy: further validation of the "modified NUTRIC" nutritional risk assessment tool. Clin Nutr. 2016;35(1):158-62.[6]
